# COPD exacerbation severity and frequency is associated with impaired macrophage efferocytosis of eosinophils

**DOI:** 10.1186/1471-2466-14-112

**Published:** 2014-07-09

**Authors:** Osama Eltboli, Mona Bafadhel, Fay Hollins, Adam Wright, Beverley Hargadon, Neeta Kulkarni, Christopher Brightling

**Affiliations:** 1 Institute for Lung Health, NIHR Respiratory Biomedical Research Unit, Department of Infection, Immunity & Inflammation, University of Leicester, Leicester, UK; 2 Department of Medicine, Faculty of Medicine, Benghazi University, Benghazi, Libya; 3 Department of Respiratory Medicine, Nuffield Department of Medicine, University of Oxford, Old Road Campus, Oxford, UK; 4 Department of Respiratory Medicine, University Hospitals of Leicester NHS Trust, Leicester, UK

**Keywords:** COPD, Eosinophils, Efferocytosis, Exacerbation

## Abstract

**Background:**

Eosinophilic airway inflammation is observed in 10-30% of COPD subjects. Whether increased eosinophils or impairment in their clearance by macrophages is associated with the severity and frequency of exacerbations is unknown.

**Methods:**

We categorised 103 COPD subjects into 4 groups determined by the upper limit of normal for their cytoplasmic macrophage red hue (<6%), an indirect measure of macrophage efferocytosis of eosinophils, and area under the curve sputum eosinophil count (≥3%/year). Eosinophil efferocytosis by monocyte-derived macrophages was studied in 17 COPD subjects and 8 normal controls.

**Results:**

There were no differences in baseline lung function, health status or exacerbation frequency between the groups: A-low red hue, high sputum eosinophils (n = 10), B-high red hue, high sputum eosinophils (n = 16), C-low red hue, low sputum eosinophils (n = 19) and D- high red hue, low sputum eosinophils (n = 58). Positive bacterial culture was lower in groups A (10%) and B (6%) compared to C (44%) and D (21%) (p = 0.01). The fall in FEV_1_ from stable to exacerbation was greatest in group A (ΔFEV_1_ [95 % CI] -0.41 L [-0.65 to -0.17]) versus group B (-0.16 L [-0.32 to -0.011]), C (-0.11 L [-0.23 to -0.002]) and D (-0.16 L [-0.22 to -0.10]; p = 0.02). Macrophage efferocytosis of eosinophils was impaired in COPD versus controls (86 [75 to 92]% versus 93 [88 to 96]%; p = 0.028); was most marked in group A (71 [70 to 84]%; p = 0.0295) and was inversely correlated with exacerbation frequency (r = -0.63; p **=** 0.006).

**Conclusions:**

Macrophage efferocytosis of eosinophils is impaired in COPD and is related to the severity and frequency of COPD exacerbations.

## Background

Chronic obstructive pulmonary disease (COPD) is a heterogeneous condition exemplified by the identification of a subgroup of COPD subjects with eosinophilic airway inflammation [1,2]. The role of eosinophilic inflammation in COPD remains controversial, but is consistently reported in 10-30% of COPD subjects and is associated with better responses to inhaled and oral corticosteroids [3,4]. The relationship between eosinophilic airway inflammation, clearance of these cells and clinical outcomes in COPD is poorly understood.

Apoptosis and subsequent removal of dead cells by phagocytes is a critical mechanism for the non-inflammatory clearance of granulocytes, including eosinophils [5-8]. Failure of phagocytosis and efferocytosis, the clearance of apoptotic cells, leads to secondary necrosis of these cells and release of toxic intracellular pro-inflammatory mediators. Impaired phagocytic ability of macrophages is consistently observed in COPD [9-14] and asthma [15], but whether this extends to abnormal efferocytosis of eosinophils in COPD needs to be determined.

Efferocytosis of eosinophils by macrophages can be measured directly *in vitro* and indirectly *in vivo* by the assessment of macrophage cytoplasmic red hue analysed on stained sputum cytospins [16]. In asthma increased macrophage cytoplasmic red hue predicts future risk of the emergence of a sputum eosinophilia and poor asthma control following corticosteroid withdrawal [16]. Whether this biomarker can identify clinically important subgroups with impaired eosinophil efferocytosis in COPD is unknown.

We hypothesised that i) COPD subjects categorised into subgroups determined by their sputum eosinophilia and sputum macrophage red hue will identify important differences in terms of their clinical characteristics, exacerbation frequency and severity, ii) macrophage efferocytosis of eosinophils in COPD will be impaired; directly related to the sputum macrophage cytoplasmic red hue and indirectly associated with exacerbation frequency and severity. To test our hypotheses, we have examined sputum cytospins available from an earlier study [17] and prospectively assessed macrophage efferocytosis in subjects that participated in this study.

## Methods

### Subjects and study design

Clinical data and sputum cytospins were available from 196 subjects that had participated in an observational study of COPD exacerbations [17,18]. Subjects had undergone extensive clinical characterisation including clinical history, demographics, visual analogue symptom (VAS) scores, health status assessment using the chronic respiratory questionnaire (CRQ) and St George’s Respiratory questionnaires (SGRQ), spirometry before and after administration of a short-acting bronchodilator, sputum analysis for cellular profiles and microbiological assessment at baseline, 3 monthly stable follow-up visits and exacerbations for at least one year. All COPD included subjects were either ex or current smokers. Subjects assessed at ≥2 stable visits with sputum cytospins of adequate quality to assess the cytoplasmic red hue of ≥50 macrophages were included.

**Figure 1:**
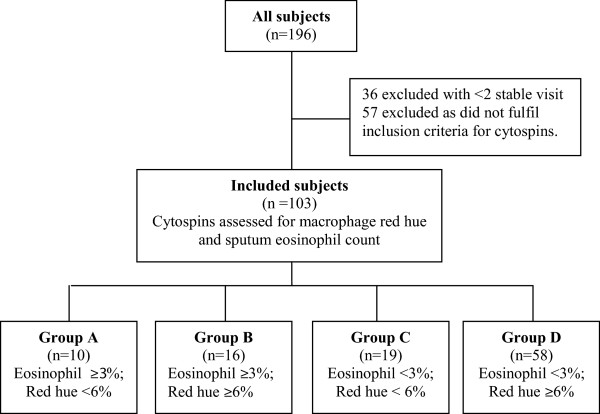
Consort diagram showing the design of the study.

From the original cohort of 196 subjects 103 subjects met the inclusion criteria (Figure 1). These subjects were not significantly different from the 196 in terms of lung function, symptoms or health status. We then imaged between 70–100 macrophages for each subject in Romanowsky-stained sputum cytospin slides, except in 15 subjects that had fewer macrophages in which at least 50 were imaged. The percentage area of cytoplasm with red hue was determined by thresholding. Using Image J software, the cytoplasmic area of macrophages is selected in saved (tiff) images (Additional file 1: Figure S1, please see additional online file 1). After defining the suitable threshold, the software calculates the number of red pixels which correspond to eosinophilic staining and the median percentage area of cytoplasm was derived as previously described [16]. The sputum eosinophil area under the curve (AUC) was derived from the sputum samples collected at stable visits and expressed as sputum eosinophil %/year. Subjects were stratified into 4 groups based on cut-offs for the sputum eosinophil count (≥3%) and the upper limit of the normal range for % area macrophage red hue (>6%) [16], (Figure 1 and Tables 1 and 2).

**Table 1:** Baseline characteristics of 4 groups of COPD patients based on their red hue of macrophages and AUC of sputum eosinophil %/year

	**A (n = 10) High sputum eosinophils, low red hue**	**B (n = 16) High sputum eosinophils, high red hue**	**C (n = 19) Low sputum eosinophils, low red hue**	**D (n = 58) Low sputum eosinophils, high red hue**	**p value**
**Male, n (%)**	6 (60)	13 (81.25)	8 (42.1)	42 (72.4)	0.06
Age (years)*	64 (3.3)	67 (2.3)	65 (2.4)	70 (1.2)	0.17
**Body mass index (kg/m**^**2**^**)**	26 (23–28)	23 (20–26)	25 (23–33)	27 (23–30)	0.09
**Pack years smoked**	49 (36–58)	47 (25–66)	47 (34–60)	48 (32–64)	0.96
**COPD duration (years)**	4.7 (2.9-10)	8.5 (2.0-12)	4.8 (2.1-6.7)	4.9 (2.8-8.1)	0.67
**Exacerbations/yr**	0.7 (0–1.8)	0.8 (0.1-1.4)	1.2 (0–1.9)	0.8 (0–1.6)	0.51
**ICS dose (BDP equivalent) (mcg/day)**	2000 (2000–2000)	2000 (1000–2000)	2000 (800–2000)	1300 (800–2000)	0.26
**FEV**_**1**_**% predicted, (%)**	62 (43–75)	44 (32–58)	43 (26–67)	55 (42–71)	0.16
**FEV**_**1 **_**post bronchodilator (L)**	1.7 (1.2-1.9)	1.1 (0.91-1.6)	0.87 (0.73-1.6)	1.4 (1–1.9)	0.13
**Post FEV**_**1**_/FVC (%)*	55 (5.4)	50 (3.3)	51 (4.2)	53 (1.6)	0.73
**Reversibility, %**	3.5 (-7.1- 8)	4.3 (1.5- 13)	4.5 (-2.2- 14)	1.5 (-4.4- 7.5)	0.33
**GOLD 1, n (%)**	1 (10)	1 (6)	5 (26)	8 (14)	0.38
**GOLD 2, n (%)**	7 (70)	6 (38)	3 (16)	26 (45)	**0.02**
**GOLD 3, n (%)**	0 (0)	7 (44)	4 (21)	19 (33)	**0.02**
**GOLD 4, n (%)**	2 (20)	2 (13)	7 (37)	5 (9)	**0.05**
**Sputum TCC ×10**^**6 **^**/g**	1.2 (0.41-2)	5.3 (2.1-8.2)	1.6 (0.94-4.5)	3.4 (1.7-7.6)	**0.002**
**Sputum neutrophils (%)**	45 (6.3)	59 (6.1)	66 (5.3)	63 (2.7)	0.07
**Sputum eosinophils (%)**	9 (1.6-35)	14 (4.9-24)	0.5 (0.25-2.8)	1 (0.3-2.1)	**<0.0001**
**Sputum eosinophil AUC (%/yr)**	6.8 (4.2-11)	5.9 (4.2-8.3)	0.5 (0.2-1.6)	0.78 (0.3-1.5)	**<0.0001**
**% of red hue of macrophages**	2.3 (1.6-3.7)	27 (11–35)	3.4 (1.8-5.3)	16 (9.1-27)	**<0.0001**
**Blood neutrophils (×10**^**9 **^**/L)**	5.1 (3.5-5.8)	4.8 (3.8-5.9)	5.9 (4.7-6.3)	5.3 (3.9-6.1)	0.13
**Blood eosinophils (×10**^**9 **^**/L)**	0.38 (0.24-0.46)	0.41 (0.26-0.56)	0.16 (0.09-0.25)	0.18 (0.12-0.30)	**<0.0001**
**CRP (mg/dl)**	5 (2.5-5.3)	5 (<5-7)	5 (2.5-11)	5 (<5-11)	0.08
**Bacterial colonisation, n (%)**	1 (10)	1 (6)	8 (44)	12 (21)	**0.04**
CFU unit/ml**	6.2 (5.8-6.6)	6.1 (5.5-6.7)	6.6 (6.3-7)	6.2 (5.9-6.4)	0.19
**MRC Dyspnoea Scale**	3 (2–4)	3 (2–4)	3 (2.5-4.5)	3 (2–4)	0.82
**SGRQ symptoms**	61 (37–80)	65 (29–82)	73 (55–84)	62 (50–77)	0.34
**SGRQ activity**	76 (35–89)	73 (48–85)	73 (59–92)	67 (48–86)	0.55
**SGRQ impacts**	31 (16–58)	44 (20–66)	42 (29–47)	32 (23–51)	0.86
**SGRQ total score**	50 (30–67)	55 (32–71)	57 (49–65)	50 (37–65)	0.75
**CRQ emotional functioning**	4.7 (3.4-5.8)	4.3 (3.9-6.3)	4.4 (3.9-5.3)	4.5 (3.6-5.6)	0.83
**CRQ fatigue**	3.8 (3.4-4.9)	3.3 (2.8-5.3)	4.0 (2.3-4.8)	3.5 (2.5-4.8)	0.51
**CRQ dyspnoea**	4 (3–4.9)	3.4 (2.6-4.2)	3 (2.2-4)	3.4 (2.2-4.4)	0.28
**CRQ mastery**	5.4 (4.4-6.4)	5.0 (3.8-6.3)	4.0 (3.8-5.5)	5.3 (3.7-6.1)	0.63
**CRQ total score**	4.4 (3.7-5.2)	4.1 (3.4-4.9)	4.0 (3.1-4.6)	4.2 (3.1-5.0)	0.50
**VAS cough (mm)**	17 (10–43)	35 (14–52)	47 (18–62)	34 (13–54)	0.23
**VAS dyspnoea (mm)**	40 (4.8-59)	43 (32–81)	51 (35–62)	46 (22–65)	0.40
**VAS sputum production (mm)**	15 (4.5-42)	36 (19–52)	38 (93–66)	28 (12–63)	0.40
**VAS sputum purulence (mm)**	14 (2–61)	17 (1–45)	38 (15–62)	21 (9.5-41)	0.41

Data presented as median (IQR) unless stated, *mean (SEM), **Geometric mean of Log data (95% CI). SEM: standard error of mean, CI: confidence interval. *Abbreviations:AUC* Area under the curve, *BDP* Beclometasone dipropionate, *CFU* Colony forming unit, *CRP* C-reactive Protein, *CRQ* Chronic Respiratory Questionnaire, *FEV*_*1*_ Forced expiratory volume in 1 second, *FVC* Forced vital capacity, *GOLD* Global Initiative for Chronic Obstructive Lung Disease, *ICS* Inhaled corticosteroids, *MRC* Medical Research Council, *SGRQ* St George’s Respiratory Questionnaire, *VAS* Visual analogue score, yr Year.

**Table 2:** Change in health status and symptom scores of 4 the groups of COPD patients at exacerbations compared to stable visits

	**A (n = 10)**	**B (n = 16)**	**C (n = 19)**	**D (n = 58)**	**p value**
**Δ CRQ Fatigue (unit)**	-1.36 (-2.61 to -0.12)*	-1.33 (-2.35 to -0.31)*	-1.11 (-1.76 to -0.45)*	-1.00 (-2.61 to -0.12)*	0.78
**Δ CRQ Dyspnoea (unit)**	-1.49 (-2.92 to -0.05)*	-1.05 (-2.32 to 0.21)*	-1.04 (-1.41 to -0.67)*	-0.57 (-0.98 to -0.15)*	0.34
**Δ CRQ Emotion (unit)**	-1.22 (-1.52 to -0.91)*	-1.07 (-2.00 to -0.14)*	-0.62 -1.27 to 0.02)*	-0.89 (-1.31 to -0.46)*	0.67
**Δ CRQ Mastery (unit)**	-0.99 (-2.33 to 0.36)*	-0.97 (-2.28 to 0.34)*	-1.10 (-1.99 to -0.21)*	-0.75 (-1.09 to -0.41)*	0.59
**Δ CRQ total (unit)**	-1.26 (-2.21 to -0.32)*	-1.12 (-1.99 to -0.22)*	-0.97 (-1.49 to -0.44)*	-0.79 (-1.04 to -0.55)*	0.54
**Δ VAS cough (mm)**	22 (3 to 42)*	35 (21 to 48)*	7 (3 to 42)*	27 (18 to 36)*	0.06
**Δ VAS dyspnoea (mm)**	34 (3 to 65)*	35 (20 to 51)*	17 (-2 to 35)	25 (17 to 33)*	0.31
**Δ VAS sputum production (mm)**	8 (-32 to 49)	24 (-2 to 50)	9 (-2 to 21)	21 (12 to 29)*	0.45
**Δ VAS sputum purulence (mm)**	28 (-13 to 68)	18 (-5 to 42)	31(17 to 45)*	15 (5 to 24)*	0.36

Data presented as mean (95% CI) unless stated; *p < 0.05 stable versus exacerbation.

To study macrophage efferocytosis, we recruited prospectively 17 of the 103 COPD subjects and 8 healthy controls. These subjects underwent clinical characterisation and donated blood to generate monocyte derived macrophages (MDM). Of the remaining subjects from the 103, 19 had died, 8 were too unwell, 20 were participating in other studies, and 39 were lost to follow-up. A further 17 subjects were recruited as peripheral blood eosinophil donors and these included 4 healthy atopic subjects and 13 subjects with asthma.

All subjects gave written informed consent and the study was approved by the Leicestershire, Northamptonshire and Rutland local ethics committee.

### Generation of monocyte–derived macrophages (MDM)

Purification of monocytes from peripheral blood was performed using Ficoll-Paque (GE Healthcare, Sweden) density gradient centrifugation and immunomagnetic positive selection of CD14^+^ monocytes using MS columns and CD14 microbeads (Miltenyi Biotec, UK) The % median [IQR] purity was 97 [88–97]% as assessed by flow cytometry using CD14-Alexa Fluor®647 conjugated antibody (Biolegend, London, UK) on a Becton Dickinson FACS Canto II. The monocytes were incubated for 6 days at 37°C and 5% CO^2^ with Dulbecco modified Eagle medium (DMEM) with high glucose, L-glutamine, D-glucose, HEPES (4-(2-hydroxyethyl)-1-piperazineethanesulfonic acid), without sodium pyruvate, phenol red (Fisher Scientific, UK) and supplemented with 10% foetal calf serum, 1% Non-essential amino acids, 1% mixture of antibiotics and antifungal (10,000 units/mL of penicillin, 10,000 μg/mL of streptomycin), and 25 μg/mL of amphotericin B) and 1% Sodium pyruvate) in the presence of recombinant human macrophage colony-stimulating factor (100 ng/ml) (R&D Systems, Europe).

### Eosinophils purification and induction of apoptosis

Eosinophils were immunomagnetically purified from peripheral blood 2 days before co-culture with MDM as described previously [16] (purity % median [IQR] was 98.1 [98.0-98.3]%). Apoptosis was induced by aging in culture deprived of cytokines for 48 hours at 37°C and 5% CO^2^. Percentages of apoptotic/dead cells were determined using Annexin V and Propidium iodide staining (BD Biosciences, USA) and flow cytometry. The percentage apoptosis within the first 2 hours after purification was <5% and after 48 hours, it was 97% [85–99] (median [IQR]).

### MDM efferocytosis of eosinophils

Apoptotic eosinophils were added to MDM in 1:5 ratio and incubated for 120 minutes in the same medium and conditions used to culture MDM [16]. Cells were then fixed and permeablised with 4% paraformaldehyde and 0.1% saponin. Immunofluorescence staining was carried out as previously described [16] with mouse monoclonal anti-human ECP (Diagnostic Development, Sweden) indirectly conjugated with RPE (Dako, Denmark) and directly conjugated CD68-FITC (Dako). Efferocytosis was quantified in 100 macrophages per donor and the percentage of MDM that had ingested fully or were engulfing eosinophils was recorded. Cytospins were prepared from the efferocytosis experiments and stained as per the sputum slides and % area of MDM red hue was measured before and after feeding with eosinophils to validate that red hue increases after ingestion of eosinophils. All the slides were assessed by a single blinded observer. The observer was blinded during the counting of MDM efferocytosis from the captured images.

### Statistical analysis

GraphPad Prism version 6 (GraphPad, San Diego) and IBM SPSS version 20 (SPSS, Inc. Chicago) were used to perform statistical analysis. Mean (standard error of the mean [SEM]) was used to present parametric data whilst median (interquartile ranges [IQR]) was used for non-parametric data and geometric mean (95% confidence interval) for data that was log normally distributed. Comparisons between groups used unpaired T-test or Mann–Whitney for parametric or non- parametric data respectively. Comparisons across groups were assessed by one-way analysis of variance (ANOVA) with Tukey pair-wise comparisons or Kruskal-Wallis test with Dunn’s multiple pair-wise comparisons for parametric and non-parametric data respectively. Chi-square or Fisher’s exact test as appropriate were used to assess categorical data. Spearman rank correlation coefficients were used to assess the correlations. A p value less than 0.05 was considered statistically significant.

## Results

**Figure 2:**
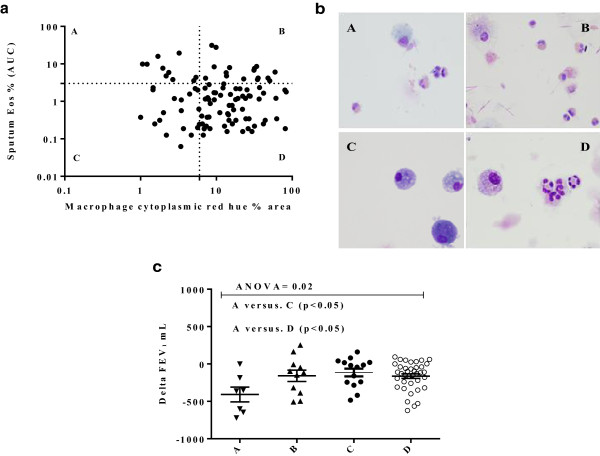
**Cytoplasmic macrophage red hue as an indirect measure of macrophage efferocytosis of eosinophils. a)** Percentage area of sputum macrophage cytoplasmic red hue in COPD subjects against sputum eosinophil area under the curve (AUC) %/year. The cut-off points for the upper limit of the normal ranges are as shown on the horizontal and vertical axes, 6 (16) and 3% respectively. **b)** Representative images of sputum macrophages: A: Subjects with high eosinophils ≥ 3% and low red hue <6%; B: high eosinophils ≥3% and high red hue ≥6%; C: low eosinophils <3% and low red hue <6%; D: low eosinophils <3% and high red hue >6%. Group B and D subjects have purple coloured cytoplasm in their macrophages. Group A and C have light blue cytoplasm. **c)** Change of FEV_1_ between stable and first exacerbation visits for the 4 groups. The lines represent mean (SEM).

The 103 COPD subjects were categorised into four groups: A-low red hue, high area under the curve sputum eosinophil count (n = 10), B-high red hue, high sputum eosinophils (n = 16), C-low red hue, low sputum eosinophil (n = 19), and D-high red hue, low sputum eosinophils (n = 58) (Figure 1). The distributions of the macrophage red hue and sputum eosinophils for the 4 groups are as shown (Figure 2a) and example cytospins for each group are as illustrated (Figure 2b). The baseline clinical characteristics of the groups are as shown (Table 1). There were no significant differences in age, gender, health status, symptoms, use of inhaled corticosteroids or exacerbation frequency between the 4 groups. Lung function was not significantly different between groups; however group A had a greater proportion of subjects with GOLD stage 2 and lower GOLD stage 3 than the other groups. The peripheral blood eosinophil count was elevated in groups A and B. Positive bacterial culture was lower in groups A (10%) and B (6%) (eosinophilic groups) compared to (non-eosinophilic groups) C (44%) and D (21%) (p = 0.04), although there was no difference in total bacterial colony forming units.

The change in lung function, health status and symptoms at the first exacerbation was compared to the mean of the prior stable visits. The change in FEV_1_ was greatest in group A**(**ΔFEV_1_ [95% CI] -0.41 L [-0.65 to -0.17]) compared to groups B (-0.16 L [-0.33 to -0.011]), C (-0.11 L [-0.23 to -0.002]) and D (-0.16 L [-0.22 to -0.10]; p = 0.02) (Figure 2c). Health status and symptoms worsened at exacerbation in all groups, but there were no differences between groups (Table 2).

**Figure 3:**
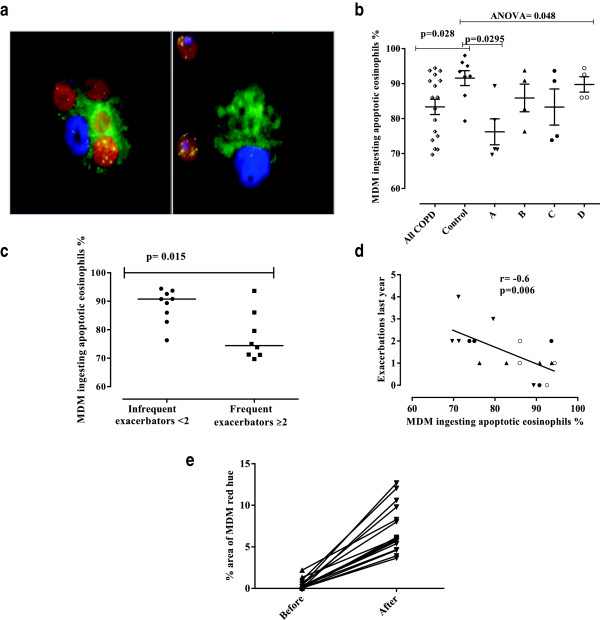
**Monocyte–derived macrophages efferocytosis of eosinophils. a)** Representative immunofluorescence staining of macrophages cultured with apoptotic eosinophils. Macrophages were stained with CD68-FITC (green), ECP-PE (red) and DAPI (blue). Left panel shows efferocytosis of eosinophils by a macrophage positively stained with ECP and the right panel shows ECP positive eosinophils and a macrophage with no evidence of efferocytosis. **b)** Comparison of macrophage efferocytosis of eosinophils in the 4 COPD groups and normal controls. The horizontal bars represent the median. **c)** Dot-plot of macrophage efferocytosis of eosinophils in those subjects with or without frequent exacerbations (≥2 exacerbations/year). Horizontal bars are medians. **d)** Scatter blot demonstrating the inverse correlation between the efferocytosis of eosinophils by macrophages and the frequency of exacerbations. Group A: triangles pointed down, B: triangles pointed up, C: closed circles, D: open circles. **e)** MDM % red hue from stained cytospins before and after eosinophil feeding for both COPD and normal subjects. Arrows represent medians of percentage area of red hue for each subject.

The clinical characteristics of the COPD subjects and healthy volunteers that provided blood to generate MDM are as shown (Table 3). There were no significant differences in age, gender, lung function, or dose of inhaled corticosteroids between the 4 COPD groups (ANOVA p = 0.26). Examples of MDM either undergoing eosinophil efferocytosis or not are shown (Figure 3a). As expected, MDM efferocytosis of eosinophils was significantly correlated with sputum macrophage cytoplasmic red hue (Spearman r = 0.54; p = 0.027). Furthermore, there was a significant increase in mean (SEM)% area of MDM red hue from 0.5 (0.2) before to 7.1 (0.7)% following COPD-derived MDM feeding with eosinophils (p < 0.0001) (Figure 3e). The mean (SEM)% area of MDM red hue after feeding with eosinophils was significantly higher in control subjects compared to those with COPD (10.3 (1.5)% versus 5.8 (0.6)%; p = 0.027).

**Table 3:** Baseline Characteristics of the COPD and healthy subjects that donated blood for the MDM eosinophil efferocytosis assays

	**COPD (n = 17)**	**Control (n = 8)**	**P value**
**Male, n (%)**	10 (58.8)	4 (50)	1
Age (years)*	68.05 (2.3)	54.1 (4.7)	0.006
**Body mass index (kg/m**^**2**^**)**	27.17 (1.36)	33 (29–34)	0.07
**Never-smokers n (%)**	0 (0)	6 (75)	0.0002
**Pack years smoked**	42 (31–70)	13 (5–22)	0.05
**COPD duration (years)**	9.6 (8–13)	NA	NA
**Exacerbations in last year**	1 (1–2)	NA	NA
**ICS dose (BDP equivalent) (mcg/day)**	2000 (1600–2000)	NA	NA
**FEV**_**1**_**% predicted, (%)**	43 (5.1)	95 (85–110)	<0.0001
**FEV**_**1 **_**post bronchodilator (L)**	1.13 (0.14)	2.8 (2.5-3.8)	0.0004
**Post FEV**_**1**_**/FVC (%)**	48.6 (4.23)	84.08 (1.7)	<0.0001
**Reversibility, %**	5.67 (2.17)	0.57 (-1.9-9)	0.45
**Sputum Total cell count ×10**^**6 **^**/g**	3.3 (1.5-6.7)	NA	NA
**Sputum neutrophils (%)**	65 (40–80)	NA	NA
**Sputum eosinophils (%)**	7.3 (4.5-15)	NA	NA
**GOLD 1, n (%)**	0 (0)	NA	NA
**GOLD 2, n (%)**	7 (41.2)	NA	NA
**GOLD 3, n (%)**	5 (29.4)	NA	NA
**GOLD 4, n (%)**	5 (29.4)	NA	NA
**Blood neutrophils (×10**^**9 **^**/L)**	4.66 (3.67-5.94)	3.5 (2.6-4.5)	0.03
**Blood eosinophils (×10**^**9 **^**/L)**	0.21 (0.08-0.36)	0.15 (0.11-0.34)	0.86
**CRP (mg/dl)**	5 (<5-8)	5 (<5-5.75)	0.95
**Bacterial colonisation, n (%)**	3 (17.6)	NA	NA
CFU unit/ml**	6.5 (5.6-7)	NA	NA
**MRC dyspnoea scale**	3 (2–4)	NA	NA
**SGRQ total score**	54 (41–66)	NA	NA
**CRQ total score**	4.1 (3.2-5.7)	NA	NA
**VAS cough (mm)**	26 (11.5-67.5)	NA	NA
**VAS dyspnoea (mm)**	50 (36–64)	NA	NA
**VAS sputum production (mm)**	23.7 (6–63)	NA	NA
**VAS sputum purulence (mm)**	12.4 (6–62)	NA	NA

Data presented as median (IQR) unless stated, *mean (SEM), **Geometric mean of Log data (95% CI). SEM: standard error of mean, CI: confidence interval.

*Abbreviations:AUC* Area under the curve, *BDP* Beclometasone dipropionate, *CFU* Colony forming unit, *CRQ* Chronic Respiratory Questionnaire, *CRP* C-reactive protein, *FEV1* Forced expiratory volume in 1 second, *FVC* Forced vital capacity, *GOLD* Global Initiative for Chronic Obstructive Lung Disease; *ICS* Inhaled corticosteroids, *MRC* Medical Research Council, *SGRQ* St George’s Respiratory Questionnaire, *TCC* Total cell count, *VAS* Visual analogue score.

The median (IQR) proportion of MDM that efferocytosed eosinophils was impaired in COPD patients 86 (75 to 92)%, compared to controls 93 (88 to 96)%, (p = 0.028) (Figure 3b). Even though the healthy controls were younger than the subjects with COPD, there was no significant difference in efferocytosis of eosinophils by macrophages between the 5 control subjects <60 years old and 3 ≥ 60 years old (p = 0.14). In addition, there was no significant correlation between MDM efferocytosis of eosinophils and age in the COPD subjects or COPD subjects and healthy controls combined. Likewise there was no relationship between smoking status or pack years and MDM efferocytosis of eosinophils in COPD subjects. There were no significant correlations between health status or symptoms and MDM efferocytosis of eosinophils. The fall in FEV_1_ at exacerbation was greater in those COPD subjects with <90% (n = 8) versus ≥90% MDM eosinophil efferocytosis (n = 5), mean delta FEV_1_ [95% confidence interval] (-22 [-5 to -38]% versus 5 [18 to -6]%; p = 0.016).The MDM efferocytosis of eosinophils was significantly different between the 4 COPD subgroups and healthy controls (Kruskal-Wallis p = 0.048). Post-hoc pairwise comparisons demonstrated that impairment of efferocytosis was greatest in group A, those subjects with high sputum eosinophils and low red hue 71 [70 to 84]% and was significantly lower than the healthy controls (p = 0.0295) (Figure 3b), but was not significantly different to the other COPD groups.

The median (IQR) proportion of macrophages that had efferocytosed eosinophils was decreased in those subjects with frequent exacerbations ≥2 compared to those with <2 exacerbation/in the last year, 74 (71 to 84)% versus 91 (84 to 93)% (p = 0.015) (Figure 3c). There was a strong inverse correlation between the exacerbation frequency in the last year and MDM efferocytosis of eosinophils (Spearman’s r = -0.60; p **=** 0.006) (Figure 3d). However, with the inclusion of the total follow-up period available prior to the efferocytosis assessment (median [IQR] follow-up period 3.2 [2.1 to 3.5] years) this correlation was lost (r = -0.29; p = 0.26). There was no correlation between GOLD stage and efferocytosis nor with red hue in the 17 COPD patients in the sub-study.

## Discussion

Here we report for the first time that macrophage efferocytosis of eosinophils is impaired in COPD and is related to increased exacerbation frequency and severity. We have assessed MDM efferocytosis of eosinophils directly *in vitro* and indirectly *in vivo* by the assessment of the sputum macrophage cytoplasmic red hue. This index of macrophage function together with the sputum eosinophil count measured over time allowed us to identify 4 subgroups of COPD segmented into those with high or low sputum eosinophil counts and high or low macrophage red hue. The group with high sputum eosinophil count and low red hue *in vivo* is predicted to represent those subjects with the greatest impairment in MDM efferocytosis of eosinophils, which was confirmed *in vitro*. This group had the greatest fall in lung function during exacerbations. Exacerbation frequency was associated with MDM efferocytosis of eosinophils and impairment was greatest in those with evidence of frequent exacerbations in the last year. Taken together our findings suggest that macrophage dysfunction in COPD might play an important role in the persistence of eosinophilic inflammation in some subjects, which in turn is related to the severity and frequency of exacerbations.

This is the first study in COPD to apply the cytoplasmic macrophage red hue, an index validated in asthma as a specific biomarker of exposure to and efferocytosis of eosinophils which had excellent interobservor and intraobservor repeatability [16]. A high cytoplasmic red hue suggests that the airway macrophages are both exposed to eosinophils and are able to competently efferocytose apoptotic cells [16]. A low red hue suggests either lack of exposure over time or impaired eosinophil efferocytosis. Indeed, macrophage red hue was correlated with MDM efferocytosis and MDM red hue increased substantially following eosinophil efferocytosis. The application of this index reveals hitherto unrecognised features of COPD. Firstly, the majority of subjects had high red hue and normal sputum eosinophil counts suggesting that the contribution of the eosinophil to the total inflammatory burden in COPD might be under-estimated. Indeed, only 18% of subjects had neither high sputum eosinophils nor high red hue. Secondly, some subjects that are exposed to eosinophils have impaired eosinophil clearance. This was confirmed by direct assessment of MDM efferocytosis of eosinophils suggesting that it is an intrinsic abnormality observed in peripheral blood derived cells rather than secondary to the airway environment. This failure of macrophage function in COPD adds to the growing evidence of impairment in macrophage efferocytosis and phagocytosis. To date this has been considered a phenomenon that promotes bacterial colonisation [9,10,12,14]. However, we have identified that impaired efferocytosis can occur in subjects with high sputum eosinophil counts and low bacterial colonisation. Therefore, a more plausible explanation is that impaired macrophage function acts as an amplification of the underlying abnormal innate immunity or inflammatory profile rather than being a primary event. This also suggests that strategies that promote macrophage function [6,19] might be best targeted at those subjects with evidence of dysfunction rather than those with bacterial colonisation *per se*.

Interestingly, the subjects that had persistently high sputum eosinophilia with a low red hue were those that had the greatest impairment in MDM efferocytosis of eosinophils and the greatest fall in FEV_1_ at exacerbations. Additionally, there was also a strong relationship between exacerbation frequency and MDM efferocytosis of eosinophils. Indeed, macrophage function was impaired in those subjects with frequent exacerbations (≥2/year) compared to those without [20]. However, this relationship did not persist retrospectively beyond the year prior to the assessment of the MDM efferocytosis, suggesting that this relationship is more variable. Future studies will need to study the stability of this relationship. Notwithstanding this potential limitation, this group might represent those that are at greatest risk and would warrant further eosinophil-specific therapy such as anti-IL-5, which would likely reduce the overall eosinophil burden as observed in asthma [21,22].

One of the strengths of our study was our ability to use data from a longitudinal observational study of COPD subjects extensively studied in stable state and at exacerbations. Although, this was a strength for the comparison of macrophage cytoplasmic red hue and sputum eosinophil counts across groups, one important criticism is that the number of subjects in each of the COPD subgroups that were recruited to study MDM efferocytosis of eosinophils was small. This was a consequence of our inability to recall many of these subjects. Additionally, although the inter- and intra-observer variability of the sputum macrophage red hue is excellent, the reproducibility of this measure and how it varies dynamically with a sputum eosinophil count overtime and in response to exacerbations is unknown and requires further study. Another potential drawback is that our normal controls were not well matched to the COPD subjects. The normal controls were younger and had a lower smoking pack year history than the COPD subjects. However, we have shown that age and smoking history, albeit in contrast to previous studies [23], were not correlated with MDM eosinophil efferocytosis. Nevertheless, further comparisons in larger populations of healthy volunteers are required to explore the effects of age upon macrophage function. The effects observed in the COPD subjects was also independent of age, therefore although age might contribute to macrophage dysfunction, we do not think it is likely to exert a major influence upon the observations we have made here. Additionally, the normal ranges for macrophage red hue were derived from our earlier work. We agree that this does not represent a large population study of the normal range of macrophage red hue, but does represent the largest study to date. Another minor potential critique is that eosinophils were obtained from subjects with asthma and or other allergic diseases. However, there was a strong correlation between red hue and efferocytosis in our study irrespective of the donor’s diagnosis. Thus, the differences observed here between health and disease are likely to be real, but need to be interpreted with caution. Together these limitations underscore the need for a larger multi-centre study, including assessment of alveolar macrophages, to validate and replicate our findings. Furthermore, we have not explored the underlying mechanisms driving the macrophage dysfunction observed here and this needs to be explored in future studies.

## Conclusions

In summary, we have found using a combination of *in vitro* and *in vivo* approaches that macrophage efferocytosis of eosinophils is impaired in COPD. The severity and frequency of COPD exacerbations was related to impaired macrophage efferocytosis of eosinophils.

## Abbreviations

COPD: Chronic obstructive pulmonary disease; FEV_1_: Forced expiratory volume one second; GOLD: Global initiative for chronic obstructive lung disease; VAS: Visual analogue symptom; CRQ: Chronic respiratory disease; SGRQ: St George’s Respiratory questionnaires; AUC: Area under the curve; MDM: Monocyte derived macrophages; IQR: Interquartile range; SEM: Standard error of mean; ANOVA: Analysis of variance; ECP: Eosinophil cationic protein; RPE: R-Phycoerythrin; IL-5: Interleukin-5; DMEM: Dulbecco modified Eagle medium; HEPES: (4-(2-hydroxyethyl)-1-piperazineethanesulfonic acid).

## Competing interests

OE MB, FH, AW, BH, NK declare that they have no conflicts of interest to disclose. CB has received grant funding and consultancy from GlaxoSmithKline, AstraZeneca, MedImmune, Roche, Novartis, Chiesi and Boerhinger-Ingelheim.

## Authors’ Contributions

OE had full access to the data and contributed to the study concept and design, recruited volunteers, performed macrophage colour assessment, phagocytosis assay, immunofluorescence, collected data, performed statistical analysis, participated in interpretation, writing and drafting of the submitted manuscript, critical review for important intellectual content and approval of the final version to be published. MB: contributed in the recruitment of volunteers, data collection, and interpretation, critical review for important intellectual content. FH: contributed to the design of the study, immunofluorescence and approval of the final version to be published. AW: contributed to the design of the study and interpretation, critical review for important intellectual content. BH: contributed in the recruitment of volunteers, data collection. NK: contributed to the design of the study, macrophage colour assessment, interpretation of data and critical review for important intellectual content. CB: contributed to the study concept and design; recruitment of volunteers, macrophage colour assessment, immunofluorescence, statistical analysis, and data interpretation, critical review for important intellectual content and approval of the final version to be published, had full access to all of the data in the study and takes responsibility for the integrity of the data and the accuracy of the data analysis and final decision to submit. All authors read and approved the final manuscript.

## Pre-publication history

The pre-publication history for this paper can be accessed here:

http://www.biomedcentral.com/1471-2466/14/112/prepub

## Supplementary Material

Additional file 1: Figure S1 Method of measuring the percentage of red hue in the macrophages by thresholding using image J software. 1, Using Image J program, a saved image of airway macrophage (TIFF file) is opened. 2, The cytoplasmic area of the macrophage is determined using the free hand-drawing tool (the forth option in the tools) as a single area without the nucleus). 3, The selection is then added to the region of interest (ROI) manager by pressing on "T" in the keyboard. In the same way the cytoplasmic areas of all the other identified macrophages in the same image are added for batch analysis (as in the shown example). 4, The plugins tool is selected, then "Macro" before clicking on "run". The red/green/blue image is converted to a hue/saturation/brightness stack. The hue image (which is seen as a grayscale image in the shown example,) is utilised to threshold for red-purple hue. 5, All the pixels with red- purple hue (those between 190–256) are identified by thresholding (shown as red-colored areas in the example, with the nucleus excluded from measurement). 6, The red hue of this area is expressed as percentage of the total measured macrophage cytoplasmic area. The same analysis is repeated for the rest of the macrophages up to one hundred macrophages per subject, and results are copied into an Excel sheet. The percentage area of red/purple hue of airway macrophage for a subject is derived by calculating the median of the red hue percentage areas of all the measured macrophages. The macro used for analysis of percentage area of red hue in macrophages (steps 4–6) was as follows: run("HSB Stack"); n = roiManager("count"); for (i = 0; i < n; i++) { roiManager("select", i); setThreshold(190, 255); run("Set Measurements…", "area area_fraction limit display redirect = None decimal = 3"); run("Measure"); updateResults()} roiManager("deselect") roiManager("Delete") run("Open Next"); roiManager("Delete") [16]. Click here for file
