# Supplementary material for: COPD exacerbation severity and frequency is associated with impaired macrophage efferocytosis of eosinophils
Source: BMC Pulm Med. 2014 Jul 9;14:112. doi: 10.1186/1471-2466-14-112 (PMC4115214; doi:10.1186/1471-2466-14-112)
Supplement: Additional file 1: Figure S1 — Method of measuring the percentage of red hue in the macrophages by thresholding using image J software. 1, Using Image J program, a saved image of airway macrophage (TIFF file) is opened. 2, The cytoplasmic area of the macrophage is determined using the free hand-drawing tool (the forth option in the tools) as a single area without the nucleus). 3, The selection is then added to the region of interest (ROI) manager by pressing on "T" in the keyboard. In the same way the cytoplasmic areas of all the other identified macrophages in the same image are added for batch analysis (as in the shown example). 4, The plugins tool is selected, then "Macro" before clicking on "run". The red/green/blue image is converted to a hue/saturation/brightness stack. The hue image (which is seen as a grayscale image in the shown example,) is utilised to threshold for red-purple hue. 5, All the pixels with red- purple hue (those between 190–256) are identified by thresholding (shown as red-colored areas in the example, with the nucleus excluded from measurement). 6, The red hue of this area is expressed as percentage of the total measured macrophage cytoplasmic area. The same analysis is repeated for the rest of the macrophages up to one hundred macrophages per subject, and results are copied into an Excel sheet. The percentage area of red/purple hue of airway macrophage for a subject is derived by calculating the median of the red hue percentage areas of all the measured macrophages. The macro used for analysis of percentage area of red hue in macrophages (steps 4–6) was as follows: run("HSB Stack"); n = roiManager("count"); for (i = 0; i < n; i++) { roiManager("select", i); setThreshold(190, 255); run("Set Measurements…", "area area_fraction limit display redirect = None decimal = 3"); run("Measure"); updateResults()} roiManager("deselect") roiManager("Delete") run("Open Next"); roiManager("Delete") [16]. [file 1471-2466-14-112-S1.docx]

**Additional: Figure S1**
